# Congenital hypothyroidism after newborn screening program reorganization in the Apulia region

**DOI:** 10.1186/s13052-022-01328-0

**Published:** 2022-07-29

**Authors:** Simonetta Simonetti, Gabriele D’Amato, Benedetta Esposito, Mariangela Chiarito, Domenico Dentico, Tania Lorè, Roberta Cardinali, Silvia Russo, Nicola Laforgia, Maria Felicia Faienza

**Affiliations:** 1 Clinical Pathology and Neonatal Screening, Azienda Ospedaliera Universitaria Policlinico-Giovanni XXIII, Bari, Italy; 2 Neonatal Intensive Care Unit, “Di Venere” Hospital, Bari, Italy; 3 Department of Biomedical Sciences and Human Oncology, Pediatric Unit, University of Bari “Aldo Moro”, Bari, Italy; 4 Department of Interdisciplinary Medicine, University of Bari “Aldo Moro”, Bari, Italy

**Keywords:** Congenital hypothyroidism, Newborn screening, Eutopic thyroid, Dysgenesis

## Abstract

**Background:**

Congenital hypothyroidism (CH) is the most frequent congenital endocrine disorder. The purpose of the present study was to evaluate the incidence and etiological classification of CH in Apulia in a three-year period according to the reorganization of the regional screening program in a single central laboratory, as well as to analyze the growth characteristics and the associated risk factors of the CH newborns diagnosed during the study period.

**Methods:**

Data derived from the reorganization of the newborn screening program for CH in a single central laboratory that collects dried blood spot (DBS) from 27 Maternity Hospitals are analyzed over a three-year period. Birth weight and length, daily dose of L-T4 at specific key points (3, 6, 12 and 18 months, 2, 2.5 and 3 years) were also obtained from medical records of the CH newborns during the study period and calculated as standard deviation score (SDS).

**Results:**

The screening program diagnosed 90 newborns with confirmed CH (incidence 1:990; recall rate: 3.6%). In detail, 75.6% newborns had an eutopic thyroid, and 24.4% had thyroid dysgenesis; 33 out of the 90 newborns (36.6%) had one or more risk factors. Among these, the multiple pregnancies are the most important because they tripled the risk of CH. At diagnosis, TSH levels were different between patients with dysgenesis and those with an eutopic thyroid (*p* = 0.005). Treatment was started at a mean of 18.5 ± 12.8 days of life. The mean starting dose of levothyroxine (L-T4) was 11.38 ± 2.46 μg/kg/day.

**Conclusions:**

The results of these study show an increase of CH cases in newborns with an eutopic thyroid compared to the traditional classification. The centralization of the screening program allows a closer cooperation between laboratory and clinical centers and facilitates the implementation of appropriate diagnostic evaluations and timely initiation of treatment, with positive effects on the management of the condition.

## Background

Congenital hypothyroidism (CH) is one of the most common endocrine diseases with onset in neonatal period, in which thyroid hormones deficiency is implicated in neuronal migration disorder and delayed psychomotor development. Most infants with CH are normal at birth and in the first two weeks of life, emphasizing the importance of the mass screening program in early diagnosis and treatment [1]. The start of Levo-thyroxine (L-T4) replacement therapy within the first weeks after birth can prevent the adverse neurological outcomes in CH newborns.

Although screening program for CH was introduced several years ago, the optimal screening method is still debated. The adoption of lower thyroid-stimulating hormone (TSH) cutoff levels [2, 3] together with the increased survival rate of preterm and low birth weight (LBW) infants [4, 5], and the introduction of re-screening for special categories of neonates at risk of delayed TSH rise [6, 7] resulted in an increased detection of milder cases, frequently associated with an eutopic thyroid gland. Conversely, severe cases due to thyroid gland dysgenesis appear unchanged over time in their incidence [5].

In the Apulia region, since September 2016, the newborn screening program for CH has been reorganized in a single central laboratory that collects dried blood spot (DBS) from 27 Maternity Hospitals. Prior to that date, each Maternity Hospital screened for CH in its own laboratory using a radioimmunoassay (RIA) method and a different blood TSH (bTSH) cutoff (ranging from 6 to 12 μU/ml). Another issue is that not all the data from the 6 laboratories in Apulia are available in the annual report of the Italian Society for the Study of Inherited Metabolic Diseases and Neonatal Screening (SIMMESN).

After the screening program reorganization, the central laboratory introduced a bTSH cutoff level of 16.0 μU/ml, with borderline results between 6.5 μU/ml and 16.0 μU/ml for the first sample, and between 5 μU/ml and < 16.0 μU/ml after the first week of age, according with the European consensus guidelines on CH [6, 7].

The aim of this study was to evaluate the incidence and the etiological classification of CH in Apulia in a three-year period corresponding to the reorganization of the regional screening program.

We also retrospectively analyzed the growth characteristics and the associated risk factors of the newborns diagnosed with CH in the study period.

## Methods

### Screening methodology

**Fig. 1:**
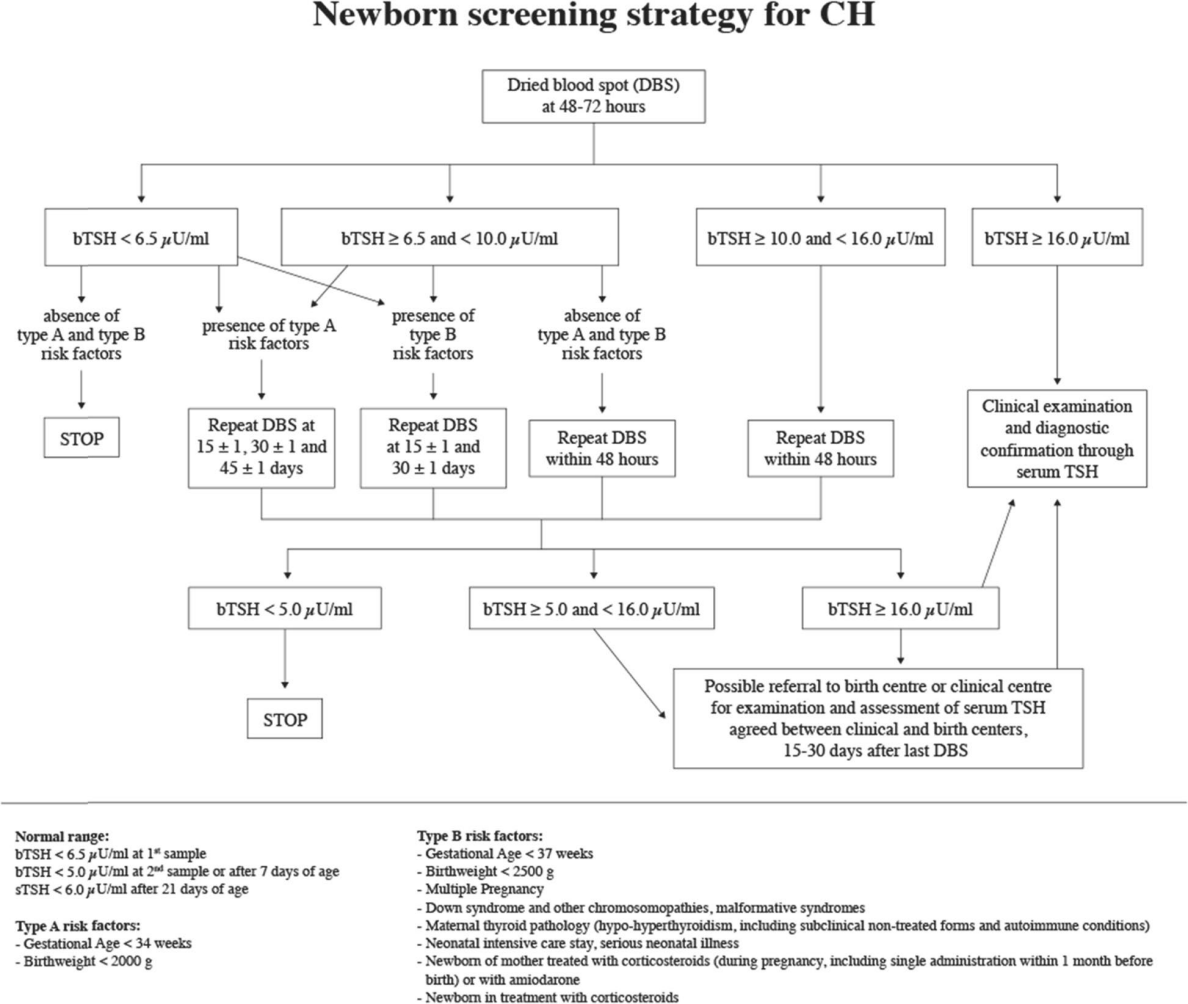
Strategy of CH screening adopted in the Regional Centre for Newborn Screening in Apulia region

The Regional Centre for Newborn Screening in Apulia uses an Automated Analyzer Genetic Screening Platform. The kit is a FluoroImmuno Assay (FIA) using the proprietary Delfia Technology (Dissociation Enhancement Lanthanide Fluoro ImmunoAssay), trademark of Perkin Elmer Inc. The sample is collected by the medical staff of each Maternity Hospital and sent to the Regional Centre for Newborn Screening. This Centre is a quality certified laboratory, with periodic quality control verification by the Biomedical Research Centre of “Azienda Ospedaliera Universitaria” in Padua. bTSH measurements are obtained using GSP Neonatal hTSH assay (manufactured by Wallac Oy, Turku, Finland), a solid phase, two-site fluoroimmunometric assay based on the direct sandwich technique. The screening strategy adopted in the laboratory is illustrated in Fig. 1.

This strategy identifies special categories of newborns, including preterm birth, low birth weight, multiple pregnancies, serious neonatal illness, maternal thyroid disorders, chromosomopathies, neonatal or maternal treatment with corticosteroids, in which the screening must be repeated more than once [8, 9].

### Clinical data

Data on birth weight and length, daily dose of L-T4 at specific key points (3, 6, 12 and 18 months, 2, 2.5 and 3 years) were obtained from medical records of the CH newborns during the study period and calculated as standard deviation score (SDS) according with the WHO standard growth charts [10].

### Statistical analysis

The t-student test for paired data was applied to compare lengths, weights, and daily doses of L-T4 in each of the key points considered for the study. The Mann–Whitney U test was used to compare TSH levels at screening and at diagnostic confirmation. The correlation coefficients were determined by the Pearson’s test. Linear regression analysis was applied to investigate linear dependence between growth-related parameters (mean length, height, and growth velocity) and average daily dose of L-T4. Data were expressed as mean ± standard deviation (SD). Sigma Plot Software 12.0 for Windows was used for data analysis, with *p* ≤ 0.05 being considered statistically significant.

## Results

During the study period (2017–2019), CH screening was performed in 89,130 newborns. Among them, 3239 had a bTSH ranging from 6.5 μU/ml and 16.0 μU/ml, thus a second DBS was done. CH was confirmed in 90 subjects (0.10%), 46 females (51.2%) and 44 males (48.8%) by evaluation of both serum TSH (sTSH) and serum FT4 (sFT4) levels, with an incidence of 1:990 live births.

Among the 90 subjects with confirmed CH, 38 had a bTSH value > 16 μU/ml, and 52 showed a bTSH value < 16 μU/ml, at the first screening.

After the confirmation of CH, the ultrasound evaluation of thyroid gland was performed in all subjects, and an eutopic thyroid gland was found in 68 newborns (75.6%), while thyroid dysgenesis only in 22 patients (24.4%). Among newborns with dysgenesis, thyroid hypoplasia was found in 13 (59%), athyreosis in 6 (27.3%), and an ectopic gland in 3 (13.7%).

### Clinical findings

Table 1 shows CH associated risk factors in the 90 CH newborns. Thirty-three out of the 90 newborns (36.6%) had one or more risk factors. Infants born from mothers with thyroid disease had a relative risk of developing CH of 0.19%, 2 times higher than the general population of newborns in the same period of the study; those born of multiple pregnancies had a risk of 0.27%, 2.7 times higher than the general population of newborns; preterm infants had a risk of 0.21%, 2 times higher than the general population. In our cohort, low birth weight infants had the lowest relative risk compared to the general population of newborns (0.15%, 1.5 times higher than the general population). A total of 11 out of 90 the CH newborns (12.2%) had extrathyroidal congenital malformations (6 congenital heart defects; 5 genitourinary abnormalities).

**Table 1:** Associated risk factors in 90 CH newborns

Risk factors	Number of newborns
Preterm Birth (GA < 37 weeks)	16/90 (17.7%)
Birth weight < 2500 g	16/90 (17.7%)
Maternal thyroid disease	10/90 (11.1%)
Multiple pregnancies	7/90 (7.7%)

*GA* gestational age. 12/90 (13.3%) preterm newborns with birth weight < 2500 g

The clinical and auxological characteristics of the cohort of 90 CH newborns according to the etiology are summarized in Table 2. Mean weight and length in each of the key points considered were within the range of 1 SDS compared to the general population. Weight and length were not different between CH newborns with an eutopic thyroid and those with dysgenesis, in each of the key points considered.

**Table 2:** Mean weight and length SDS according to etiology

	**Dysgenesis**			**Eutopic thyroid**
	Weight	Lenght	Weight	Lenght
At birth	-0.78 ± 2.88	-1.03 ± 2.57	-1.03 ± 1.26^a^	-0.81 ± 1.53^a^
3 months	-1.34 ± 1.9	-0.36 ± 1.29	-0.36 ± 1.46^a^	-0.65 ± 1.46^a^
6 months	0.3 ± 0.53	0.01 ± 1.04	0.01 ± 1.04^a^	-0.08 ± 0.91^a^
12 months	0.64 ± 1.19	0.48 ± 0.97	-0.48 ± 0.97^a^	0.09 ± 1.12^a^
18 months	1.33 ± 1.92	0.24 ± 1.07	-0.24 ± 1.07^a^	-0.5 ± 1.25^a^
2 years	1.54 ± 2.53	0.17 ± 0.81	0.18 ± 0.81^a^	-0.43 ± 0.96^a^
2.5 years	1.51 ± 2.78	-0.01 ± 0.92	-0.01 ± 0.92^a^	-0.09 ± 0.89^a^
3 years	0.6 ± 1.8	0.05 ± 0.9	0.05 ± 0.9^a^	-0.46 ± 0.42^a^

^a^vs dysgenesis *p* > 0.05

bTSH levels at screening and sTSH at diagnostic confirmation are shown in Table 3. bTSH median values at screening between the CH newborns with an eutopic thyroid and those with dysgenesis were not statically different (*p* = 0.13), while a significant statistical difference of sTSH median values was found at the time of diagnostic confirmation (*p* = 0.005), with higher levels in newborns with dysgenesis. bTSH median values levels at screening and sTSH levels at diagnostic confirmation were not statistically different between preterm and at term infants.

**Table 3:** bTSH levels at screening, sTSH at diagnostic confirmation, starting day of treatment

	**At term**	**Preterm**	**Dysgenesis**	**Eutopic thyroid**
bTSH at screening (µU/ml)	40.7 ± 47.3	24.5 ± 9.5^a^	98.1 ± 63.4	30 ± 30.1^b^
sTSH at diagnostic confirmation (µU/ml)	192.7 ± 235.7	58.3 ± 41.9^a^	358.1 ± 245.2	131.6 ± 174.5^c^
Starting day of treatment	17.8 ± 12.6	24.3 ± 14.4	11.6 ± 9.12	18.9 ± 11.9^**c**^

3/16 preterm newborns (18.7%) were positive at the first screening; 13/16 preterm newborns (81.3%) were positive at the second screening

^a^vs at term *p* > 0.05

^b^vs dysgenesis *p* > 0.05

^c^vs dysgenesis *p* = 0.005

An inverse correlation between the starting day of treatment and bTSH levels at screening (*r* = -0.654; *p* < 0.001) and sTHS at diagnostic confirmation (*r* = -0.525; *p* < 0.001) was observed.

The evaluation of a possible correlation between bTSH levels at screening and sTSH at diagnostic confirmation with growth-related parameters (weight and length in each of the key points considered) did not reveal a relationship of mutual interdependence between those variables. A slight statistically significant inverse correlation (*r* = -0.509) was found between sTSH levels at diagnostic confirmation and length at 2 and 2.5 years of age (*p* = 0.016 and *p* = 0.031, respectively).

Table 3 shows the mean starting day for preterm and at term newborns and according with etiology are shown. The mean starting dose of L-T4 in the cohort of 90 CH newborns was 11.38 ± 2.46 μg/kg/day; the mean starting dose of L-T4 was 11.48 ± 5.23 μg/kg/day in CH newborns with an eutopic gland and 12.39 ± 3.14 μg/kg/day in those with dysgenesis.

The mean day of starting treatment with L-T4 was different according to etiology, as newborns with dysgenesis started at 11.6 days ± 9.12 and newborns with gland in situ started at 18.9 days ± 11.9.

A significant statistical difference of the mean daily dose of L-T4 between the CH newborns with an eutopic gland and those with dysgenesis was observed at 3 months (*p* = 0.02), 18 months (*p* = 0.041), 2 years (*p* = 0.007), 2.5 years (*p* = 0.006) and 3 years (*p* = 0.045).

The correlation analysis has shown a slight inverse correlation between the mean dose of L-T4 and growth-related variables: mean weight (*r* = -0.353; *p* = 0.008); mean length (*r* = -0.301; *p* = 0.025), and mean growth velocity (*r* = -0.377; *p* = 0.013). However, linear regression analysis for length, weight and growth velocity as dependent variables and mean daily dose of L-T4 as independent variable, did not confirm the inverse correlation among these variables.

## Discussion

This study is a comprehensive report of our experience with the reorganization of the newborn screening for CH in Apulia region over a three-year period. The overall incidence of 1:990 we found resulted in line with the national mean incidence of IC, although an extreme variability of the Italian incidence of IC due to inter-regional variations and within each region for the presence of several laboratories has been reported in the SIMMESN annual report.

The most important change in the reorganization program is that a second DBS at 15 days of life was performed in those newborns at risk for delay of TSH increase, according to European guidelines [6, 7]. Another important finding of our study is a marked increase in CH cases with a normally located gland, which account for almost 3/4 of all confirmed cases within the population examined. Our data are consistent with other epidemiological studies [11, 12] which revealed an increasing incidence of CH forms of mild and moderate entity, which are often re-evaluated as transient CH after the first 3 years of life and are mostly characterized by an eutopic thyroid gland. This observation is due both to the adoption of lower THS cutoff levels, and to increased survival of preterm infants screened several times to diagnose a possible delayed rise of TSH [2].

In our study population more than 1/3 of 90 CH newborns had at least one risk factor, requiring repeating screening test, in accordance with the strategy established by the Regional Centre for Newborn Screening. Multiple pregnancies are the most significant relative risk factor because the risk of CH is tripled, in agreement with previous data [13].

The evidence of higher TSH levels at diagnostic confirmation in newborns with dysgenesis compared to subjects with an eutopic gland suggests the importance of reorganizing the screening program, because the adoption of lower TSH cutoffs levels allows to diagnose a significant number of mild CH cases with an eutopic thyroid largely undiagnosed in the past [14]. As expected, CH newborns show an increased prevalence of other congenital malformations, compared to general population [15], suggesting a common genetic component for CH and other congenital defects [16].

The longitudinal analysis of growth-related parameters confirmed that appropriately treated, CH newborns, regardless of its etiology, do not suffer of growth alterations, as already evidenced by several studies [17, 18]. Growth was not correlated to TSH levels both at screening and at diagnostic confirmation. The inverse correlation between TSH levels both at screening and at diagnostic confirmation with the starting day of treatment underlies the close cooperation between Screening Laboratory and reference Clinical Centre, essential for a correct and timely treatment of CH, as recommended by Italian guidelines [19].

Several studies have shown that an early start of replacement therapy is essential for a normal physiological neurodevelopment of CH child [20, 21]. The most recent European and Italian guidelines identify the first two weeks of age as the time to start treatment [6, 7, 19].

In our study, the mean starting day of treatment in term newborns was of 17.8 ± 12.6 according to recommendation, and over three weeks of life, at 24.3 ± 14.4 days in preterm newborns [22]. The mean starting dose of L-T4 falls within the recommended range [6, 7, 12]. The significant difference of L-T4 dose between CH newborns with dysgenesis and those with an eutopic thyroid at 3 months, 18 months, 2, 2.5 and 3 years, confirms that a different dose of L-T4 is required according to CH etiology [23–25].

One of the limitations of this study is the absence of data after the first three years of life, when a re-evaluation should be done in patients with eutopic gland.

## Conclusions

The centralization of the newborn screening program, together with a close cooperation between the central laboratory and clinical centers allows the most appropriate diagnostic evaluations and a timely initiation of treatment, according to the biochemical data, with a better management of CH a more favorable neurodevelopmental outcome in affected subjects. We have observed an increase in CH cases with an eutopic thyroid gland, changing the relative prevalence of CH etiologies compared to the past. We can confirm that CH, when adequately treated and monitored, does not influence growth in the first years of life, independently of its etiology and severity.

## Data Availability

The datasets used and/or analysed during the current study are available from the corresponding author on reasonable request.

## Abbreviations

CH: Congenital hypothyroidism
L-T4: Levo-thyroxine
DBS: Dried blood spot
SDS: Standard deviation score
TSH: Lower thyroid-stimulating hormone
LBW: Low birth weight
RIA: Radioimmunoassay
FIA: Fluoroimmunoassay
bTSH: Blood TSH
sTSH: Serum TSH
